# In Situ Photoresin Synthesis via Reactive Diluents for Vat Photopolymerization

**DOI:** 10.1021/acs.biomac.5c01471

**Published:** 2025-10-09

**Authors:** Tao Zhang, Vincent S. D. Voet, Rudy Folkersma, Katja Loos

**Affiliations:** † Macromolecular Chemistry and New Polymeric Materials, Zernike Institute for Advanced Materials, 3647 University of Groningen, Nijenborgh 3, Groningen, 9747 AG, The Netherlands; ‡ Circular Plastics, Academy Tech and Design, 84808 NHL Stenden University of Applied Sciences, Van Schaikweg 94, 7811 KL, Emmen, The Netherlands

## Abstract

As climate change intensifies, there is a pressing demand for sustainable alternatives to fossil-derived photoresins in additive manufacturing. While biobased systems have been explored, many rely on hazardous solvents, limiting their environmental benefits. Here, we report a one-pot, purification-free strategy for synthesizing renewable, high-performance photoresins using furan-based monomers derived from lignocellulosic biomass. Furfuryl methacrylate and 4,4′-bismaleimidodiphenylmethane (BSM) were integrated into methacrylate networks via Diels–Alder (DA) chemistry, with 2-hydroxyethyl methacrylate (HEMA) enabling high conversion (93%) under optimized conditions. Mechanical testing revealed that UV postcuring enhanced tensile strength, whereas excessive UV or solvent exposure caused oligomer leaching. Thermal postcuring activated retro-DA reactions, improving mechanical robustness and shape memory performance. Comparative studies showed aromatic DA derivatives offered superior programmability, while aliphatic analogs provided higher renewable carbon content with stable printability. This scalable, solvent-free strategy establishes a green chemistry framework for sustainable, high-performance photoresins, advancing additive manufacturing toward circular economy objectives.

## Graphical abstract

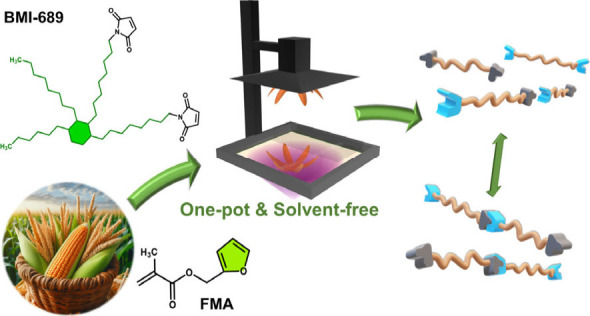

## Introduction

The pursuit of environmentally sustainable photopolymer systems for additive manufacturing continues to face significant challenges, particularly regarding the widespread use of hazardous solvents and multistep synthetic protocols.^1^ Despite the growing interest in biobased and biodegradable monomers, most current photoresin formulations rely heavily on solvent-based methods for the synthesis and isolation of intermediates, undermining their environmental advantages.^2,3^ These processes not only involve waste-intensive purification steps but also generate volatile organic compounds, contradicting the principles of green chemistry.^4,5^ Even in recent studies in high-impact journals, the use of volatile organic solvents persists as a foundational component of resin preparation, from dispersing starting monomers to isolating final products.^6^ This not only elongates the synthesis process but also heightens safety risks and environmental exposure associated with volatile organic compounds (VOCs) emissions.^7^

To address this critical limitation, we report a solvent-free, one-pot strategy that enables the direct formation of a photoprintable resin through in situ Diels–Alder (DA) chemistry. In this method, the DA reaction between a biobased furan monomer and a bismaleimide dienophile proceeds directly within a reactive methacrylate diluent. This approach avoids the need for additional solvents or postreaction purification, achieving 100% atom economy and greatly simplifying the production process. Notably, the methacrylate diluent remains chemically inert during the DA reaction but becomes fully reactive during UV curing, enabling the formulation of a low-viscosity, photocurable resin suitable for high-resolution vat photopolymerization. Importantly, this methodology is readily extendable to other mild reactions, offering a viable strategy for green photoresin synthesis applicable to vat photopolymerization.

In addition to its synthetic simplicity, the resulting resin retains the dynamic features of the DA adducts, allowing for thermally reversible bond exchange and shape memory behavior. The use of biobased furan compounds further enhances the sustainability profile, but it is the integration of solvent-free synthesis with direct printability that defines the central innovation of this work. By shifting the focus from merely renewable feedstocks to process-level sustainability, this strategy offers a more holistic and scalable path toward green additive manufacturing.

Herein, we present a biomass-derived furan-based monomer synthesized from furfuryl methacrylate (FMA) and bismaleimide via a DA reaction (Figure 1). Poly­(ethylene glycol) methacrylate (PEGMA) and a photoinitiator are incorporated to develop photosensitive resins suitable for 3D printing. The procedure can be divided into two distinct steps. First, The DA reaction from bismaleamide and furan takes place in the presence of the diluent, which serves solely as a thinner material and does not participate itself in the reaction (Figure 1a,b). Next, the addition of photoinitiator (BAPO) facilitates the photopolymerization of the prepared mixture including the DA product and PEGMA, in the printer (Figure 1c). This strategy minimizes solvent usage while facilitating the DA reaction by addressing the challenge of high viscosity, which can impede the synthesis process. Here, two types of bismaleimides were used as reactants for the DA reaction in the photoprintable ink formulation. One of them, 4,4’-bismaleimidodiphenylmethane (BSM), a commercially available, fossil fuel-based bulk chemical that offers a cost-effective option for additive manufacturing. The second, BMI-689, is biobased a bismaleimide synthesized from fatty acid derivatives, contributing to the flexibility of resin formulations.^8^ Unlike conventional bismaleimides, BMI-689 possesses a non-hydrogenated dimer diamine backbone, which lowers its viscosity and enhances its reactivity as a dienophile in the DA reaction system. Additionally, the use of biobased building blocks further enhances the sustainability of this approach.

**Figure 1:**
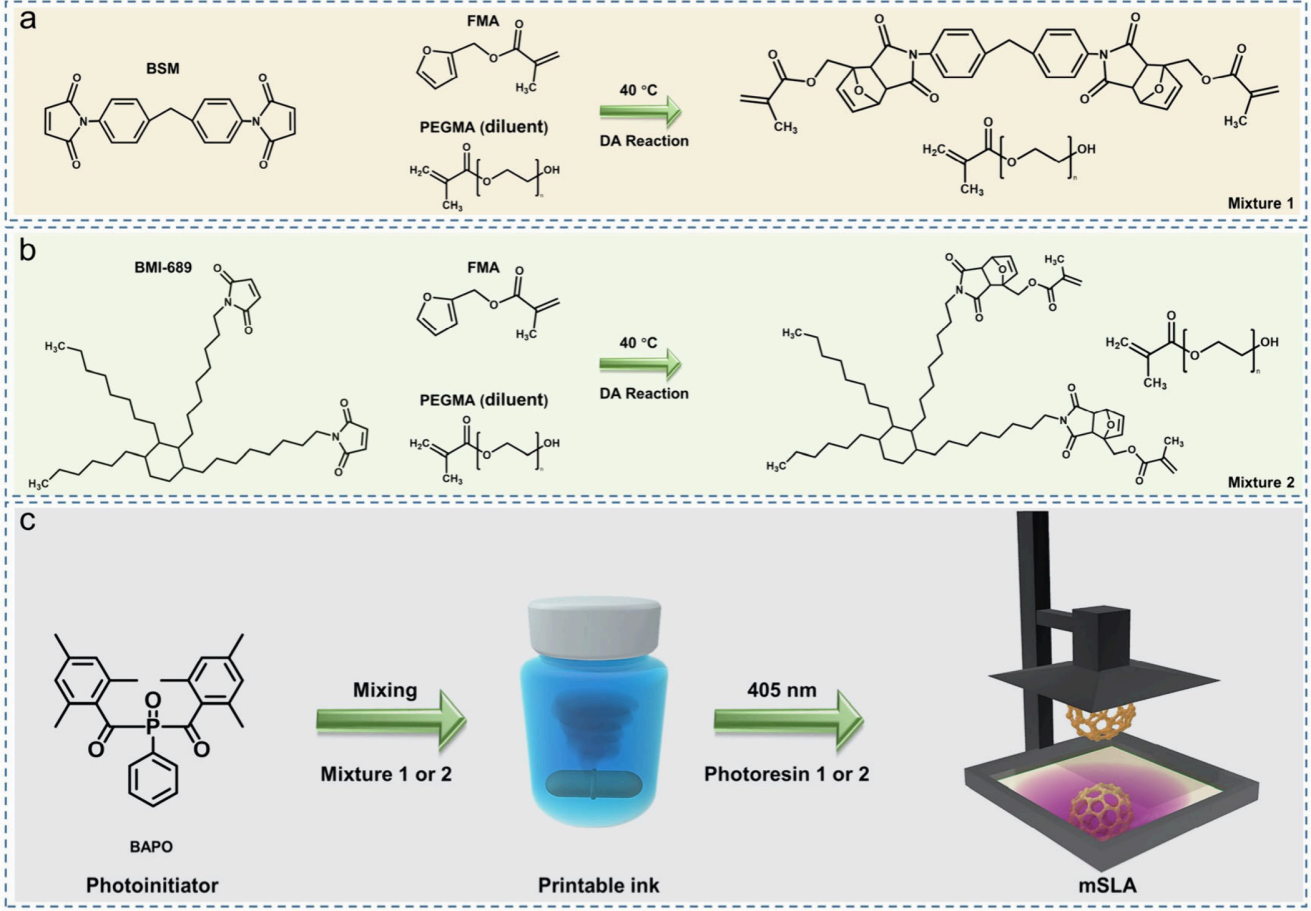
Synthesis route of the methacrylate mixtures via Diels–Alder preparation from renewable bismaleamine and furfuryl methacrylate at 40 °C for 12 h: (a) BSM as dienophile; (b) BMI-689 as dienophile; (c) Process of obtaining photoprintable resin for vat photopolymerization.

## Methods

### Materials

Methyl methacrylate (MMA, which contains ≤30 ppm MEHQ as an inhibitor, 99%), 2-hydroxyethyl methacrylate (HEMA, which contains ≤250 ppm monomethyl ether hydroquinone as an inhibitor, 97%), *tert*-butyl methacrylate (tBMA, 98%, which contains 200 ppm monomethyl ether hydroquinone as an inhibitor), lauryl methacrylate (LMA, which contains 500 ppm MEHQ as an inhibitor, 96%), poly­(ethylene glycol) methacrylate (PEGMA, average Mn 360, methacrylate, 500–800 ppm MEHQ as an inhibitor), dimethyl sulfoxide-d6 (CD3)_2_S = O, 99.5 atom % D), furfuryl methacrylate (FMA, 97%, which contains 200 ppm monomethyl ether hydroquinone as an inhibitor), and phenylbis­(2,4,6-trimethyl benzoyl) phosphine oxide (BAPO, 97%) were purchased from Sigma–Aldrich. 4,4’-Bismaleimidodiphenylmethane (BSM, > 96.0% (GC)) was obtained from TCI (Tokyo Chemical Industry, Japan), and low-viscosity liquid bismaleimide (BMI-689, 1,500 ± 500 cP) was obtained from CAPLINQ Europe BV.

### Resin Formulations

The liquid printing precursor contains bonds that can undergo Diels–Alder (DA) reactions, which are synthesized by mixing FMA with either BSM or BMI-689 at 40 °C for 12 h to form a DA-based dimethacrylate intermediate in acetone, MMA, tBMA, LMA, and PEGMA solutions, which serves as an unreactive diluent. The conversion yield was calculated based on the intensity of the new proton signal appearing at 5.2–5.4 ppm, corresponding to the cycloaddition furan. This intensity was divided by the sum of the proton signal intensity at 7.67 ppm, attributed to the unreacted furan, and the proton intensity at 5.2–5.4 ppm from the cycloaddition furan. A photoinitiator, BAPO (1 wt %), was subsequently added to produce the final photosensitive resin. The functional molar ratio of FMA to BSM or BMI-689 was maintained at 1.1:1 (FMA:BSM/BMI-689 molar ratio of 2.2:1) to achieve a high conversion rate in the DA reaction, with a PEGMA to DA-based dimethacrylate molar ratio set at 2:1, resulting in mixtures 1 and 2, respectively.

### Vat Photopolymerization

Printing was conducted via a bottom-up 3D printer (Phrozen Sonic 4K 2022) with a 405 nm ParaLED Matrix 3.0 light source with 2.5 mW/cm^2^ irradiance. The print models were prepared via the Phrozen Dental Synergy Slicer. The samples containing DA aromatics (Photoresin 1) were printed with a layer height of 100 μm and an exposure time of 8 s. In contrast, the DA-aliphatic samples (Photoresin 2) required 16 s for solidification at the same layer height of 100 μm due to variations in the methacrylate concentration within the resin. Following the printing process, the printed samples were cleansed via a paper towel moistened with isopropyl alcohol to eliminate residual resin at the surface, air-dried for 10 min, and then post-UV cured (405 nm, LED radiant of 6 W) for 30 min at ambient temperature.

### Sustainability Analysis

The sustainability metrics of entire processes were assessed using the zero and first passes of the CHEM21 metrics toolkit, as outlined by McElroy et al.^9^ Atom economy (AE), which measures the remaining atoms in the product, is calculated by the ratio of the molecular weight of the desired products to that of the reactants. Reaction mass efficiency (RME) evaluates the experimental efficiency. The E-factor, a more comprehensive metric, considers waste production and is defined as the mass ratio of waste to desired products.^4^ EcoScale, which takes into account yield, cost, safety, setup, temperature, reaction duration, workup, and purification, is deemed the most effective tool for evaluating the sustainability of these polymerization reactions.^10^

## Characterization

The ^1^H NMR spectra of the monomers and reaction kinetics of the DA-based process were acquired via a Bruker Ascend NMR400 spectrometer at 25 °C in DMSO-*d*_6_ with 16 scans.

Attenuated total reflection-Fourier transform infrared (ATR-FTIR) spectra were acquired via a Bruker VERTEX 70 spectrometer equipped with a diamond single-reflection ATR accessory and scanned over the 4000–700 cm^–1^ range. To further investigate the absorbance of the dynamic changes, an ATR accessory with a heater was used to record the spectra across a temperature range of 40 to 120 °C.

Tensile testing was performed on an Instron testing apparatus equipped with a load cell capacity of 1 kN to ascertain the mechanical characteristics of the materials under examination. The strain rate was maintained at 5 mm/min, and the samples for tensile testing were printed in a dumbbell configuration with dimensions of 48.75 mm × 3.25 mm × 1.3 mm. The measurement was repeated three times to ensure reproducibility.

The thermal stability of the cured resins was analyzed via thermogravimetric analysis (TGA) on a TA Instruments Q series with an autosampler using a temperature ramp from 25 to 700 °C at a rate of 10 °C min^–1^ under atmospheric flow.

Polarized optical microscopy (POM) was performed via a Zeiss Axiophot microscope, with the samples positioned between crossed polarizers.

X-ray diffraction patterns of the DA aromatic film were recorded using a Bruker D8 Advance apparatus. The diffractograms were collected over a scanning range of 2–60° (2θ) under 40 kV and 40 mA conditions, employing Cu Kα radiation with a wavelength of 0.1542 nm.

Glass transition temperatures (*T*_g_) were measured via a TA Instruments Discovery HR20 instrument in DMA mode (1 Hz, 0.2% strain, heating rate: 3 °C min^–1^). The test samples had dimensions of 20 mm × 5 mm × 2 mm.

The shape memory test for the bent spline was conducted using a hot plate set to 90 °C. Additionally, the shape memory of the printed follower was demonstrated by immersing it in hot water at 90 °C.

## Results

Initially, the cycloaddition between FMA and aromatic maleimide (BSM) was used as a model reaction to investigate the reaction kinetics at 40 °C. A comparison of different diluent systems revealed their impact on the reaction yield and potential failure. This is important because the choice of diluent affects not only the solubility of the products and the dispersion of reactants but also plays a crucial role in ensuring reproducibility and optimizing reaction yields in the synthesis of DA-based intermediates. Acetone, MMA, tBMA, LMA, HEMA, and PEGMA were used as diluents in the cycloaddition reaction between FMA and BSM, with the functional molar ratio set at 1:1. Table 1 shows that tBMA and LMA are unsuitable diluents because of the phase separation observed during the process. In contrast, the conversion yields for the diluents acetone, MMA, HEMA, and PEGMA were 84.6%, 84.1%, 84.7%, and 85.9%, respectively (the ^1^H NMR spectra of the corresponding products are given in the Figures S6, S7, S8 and S11, showing minimal variation). To optimize the cycloaddition yield, HEMA was selected as a reactive diluent due to its widespread use, compatibility with the reaction system, and potential for future biobased production. While currently derived from fossil resources, HEMA represents a commonly used option, and biobased methacrylate-derived diluent (for example, isobornyl methacrylate) may support greener chemistry initiatives in the future. The furan-to-maleimide functional molar ratios were adjusted to 1:1, 1.05:1, and 1.1:1. The proton signals from furan in the ^1^H NMR spectrum were monitored to calculate the yield. The results showed yields of 84.7%, 90.1%, and 93.4% for the 1:1, 1.05:1, and 1.1:1 furan-to-maleimide molar ratios, respectively (Table 1 and Figures S8–S10). These results indicate that increasing the feed ratio improves the conversion yield. This trend can be attributed to the properties of the reactants: maleimide, in the solid state, and furan methacrylate (FMA), a low-viscosity liquid that acts as both a reactive monomer and a diluent. Using excess FMA not only shifts the equilibrium to favor product formation but also facilitates better mixing and reaction efficiency due to its liquid state.

**Table 1:** Composition of the Reactants and Diluents Used for the DA Reaction

resin	diluent	FMA/BSM (functional molar ratio)	yield (%)
R1	acetone	1	84.6
R2	MMA	1	84.1
R3	tBMA	1	No
R4	LMA	1	No
R5	PEGMA	1	85.9
R6	HEMA	1	84.7
R7	HEMA	1.05	90.1
R8	HEMA	1.1	93.4

Due to the pronounced brittleness observed in printed specimens prepared with HEMA as a diluent, PEGMA was selected as an alternative diluent in subsequent experiments to facilitate comprehensive mechanical characterizations. The synthesis of the furan-based monomer is straightforward (Figure 1a,b). Initially, furan and maleimide are mixed in PEGMA, which acts as a unreactive diluent. Following the cycloaddition of furan and maleimide (both aromatic and aliphatic), the photoinitiator BAPO is added to the mixture, enabling its use in photoprinting, where PEGMA now serves as a reactive diluent. As shown in Figure 2a,b, the DA-based aromatic and aliphatic products were characterized via ^1^H NMR. The proton shift at approximately 7.67 ppm is characteristic C-5 proton of FMA, whereas the protons from 5.2 to 5.4 ppm correspond to the furan moiety after cycloaddition with bismaleimide.^11^ On the basis of the intensity variation, the conversion yield can be calculated. After the addition of a photoinitiator, a printable photoresin was obtained. The expected chemical structures of the printed (cured) resins are shown in Figure 2c,d.

**Figure 2:**
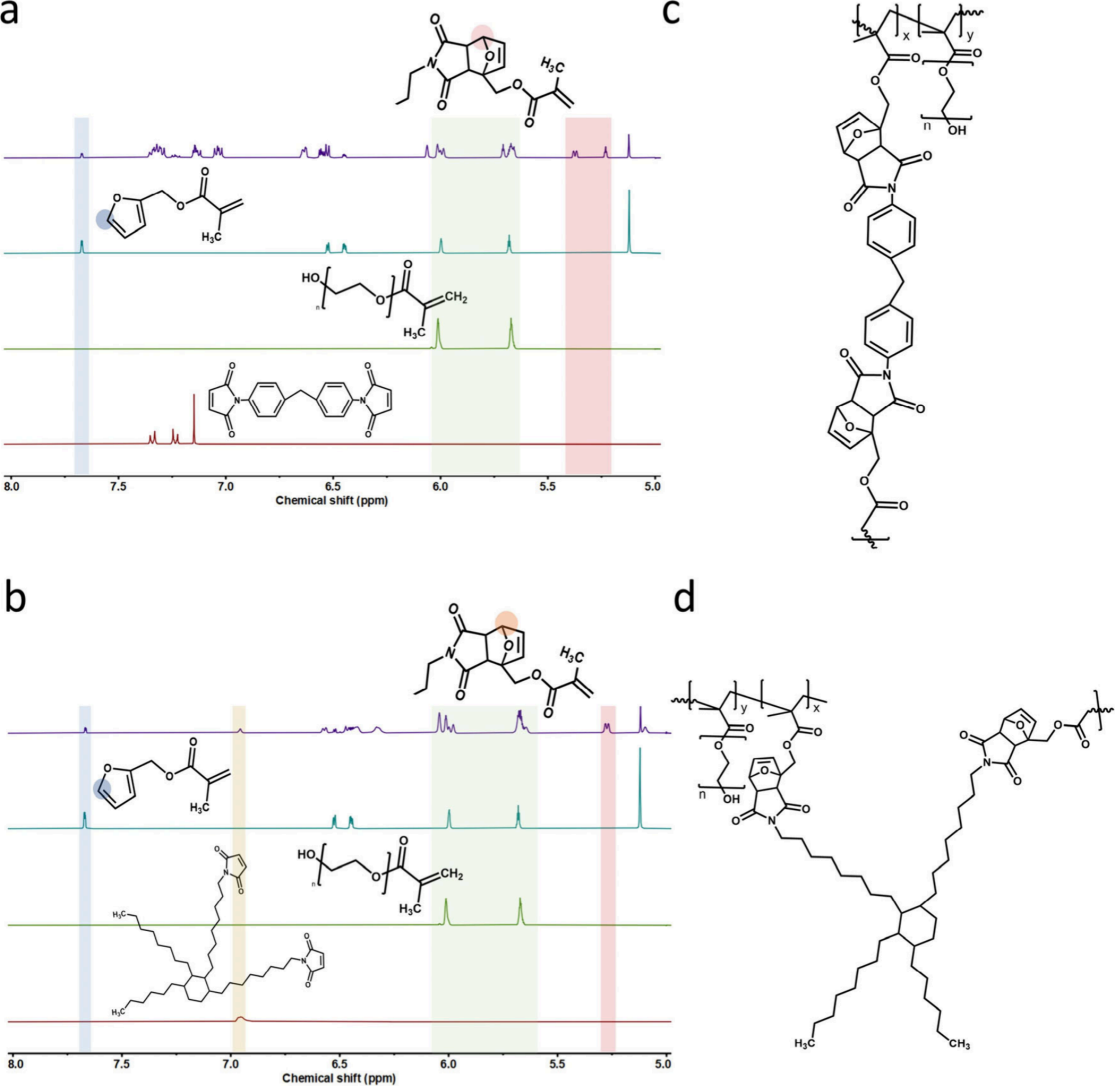
^1^H NMR spectra (400 MHz, DMSO-*d*_6_, 25 °C) of the (a) FMA, BSM, and DA aromatic intermediates; (b) FMA, BMI-689, and DA aliphatic intermediates; (c) Possible chemical structure of DA-based aromatic network after photoprinting; (d) DA-based aliphatic network after photoprinting.

We then conducted the postcuring analysis of the aromatic DA-based ink. Figure 3a shows the variation in the absorbance of the ink under different conditions. The peak at 1778 cm^–1^ corresponds to the C = O stretching vibrations in the DA adduct, confirming successful adduct formation during the preparation of Mixture 1. Upon the addition of BAPO to Mixture 1 and subsequent mSLA printing (Photoresin 1), printed splines were produced for various post-treatment processes. The increased cross-link density resulting from the DA cycloaddition leads to a more rigid and brittle polymer network, making the material hard and fragile. As the restricted mobility of polymer chains makes the materials more susceptible to fracture under stress. Therefore, using a reactive diluent with a longer molecular chain is preferable to one with a shorter chain. The results align with the expected printing outcomes, as samples printed with PEGMA as the diluent were less brittle than those printed with HEMA.

**Figure 3:**
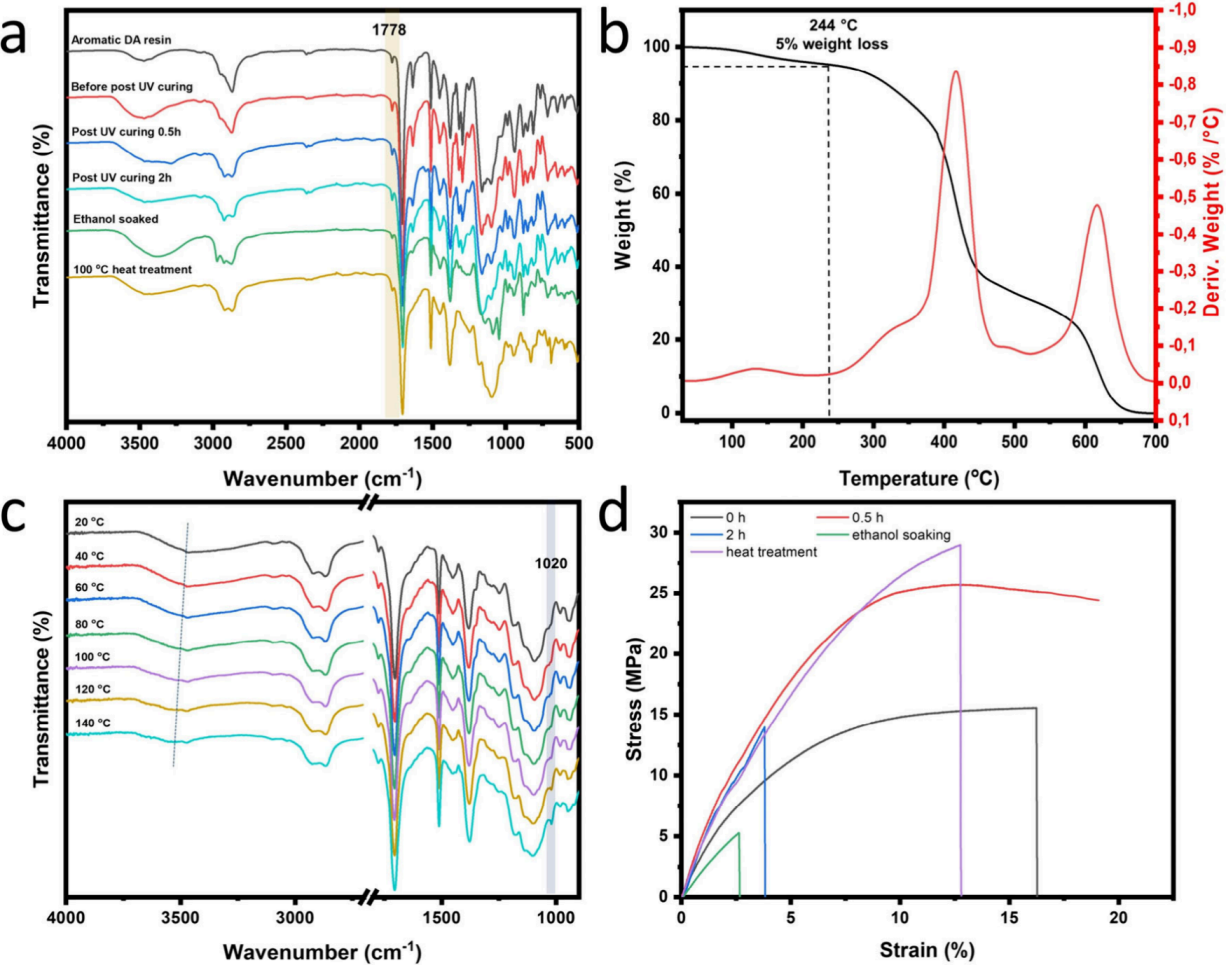
(a) ATR-FTIR spectra from 4000 cm^–1^ to 500 cm^–1^ of aromatic furan-based photosensitive ink and printed splines with different postcuring processes; (b) TGA and DTG curves of the printed samples after UV postcuring for 0.5 h; (c) ATR-FTIR spectra of the printed aromatic thin film in the temperature range of 20 °C to 140 °C; (d) Stress–strain curves of the printed films under various post-treatment conditions, including no treatment (black line), UV postcuring for 0.5 h (red line) and 2 h (blue line), ethanol soaking (green line), and heat treatment at 100 °C (purple line).

The C=O stretching vibration intensity slightly decreases after ethanol soaking, primarily due to the removal of free oligomeric DA components. Another notable peak at approximately 1635 cm^–1^, corresponding to the C=C stretching vibration, progressively diminishes following ethanol treatment and disappears entirely after UV postcuring and overnight heat treatment at 100 °C. The complete disappearance of the C=C signal confirms the full polymerization and cross-linking of the methacrylate groups. This significant reduction demonstrates that the combination of UV postcuring and heat treatment is vital for producing fully polymerized prints with minimal residual monomers, thereby ensuring enhanced mechanical and chemical stability.

To evaluate the macroscopic performance, the mechanical properties of the printed splines subjected to different post-treatments were assessed using tensile stress–strain tests. As shown in Figure 3d and Table 2, the sample without post treatment exhibits an ultimate tensile strength of 15.16 ± 1.07 MPa with a strain of 15.27 ± 1.04% before breaking. After 0.5 h of UV irradiation, the ultimate stress increases significantly, whereas the strain decreases slightly, which is due to increased cross-link density as a result of the UV exposure. The sample postcured for an additional 2 h confirmed this trend (Figure 3d), with the ultimate stress decreasing to 13.90 ± 0.29 MPa and the strain decreasing to 3.69 ± 0.13%. This decline is attributed to polymer network embrittlement caused by prolonged UV exposure. Extended irradiation can induce overcross-linking and chain scission within the polymer backbone, leading to a more brittle network with reduced molecular mobility and the accumulation of microdefects. Consequently, while moderate postcuring strengthens the material, excessive UV exposure deteriorates its mechanical performance. These observations are consistent with prior reports on UV-induced embrittlement of photopolymer networks.^12^ These results confirm that an appropriate UV postcuring time is crucial for optimizing 3D printing, as prolonged UV exposure can render the polymer network more brittle.

**Table 2:** Ultimate Stress and Strain at Ultimate Stress of the Printed DA Polymer

DA polymer type	post-treatment condition	ultimate stress (MPa)	strain (%)
	no	15.16 ± 1.07	15.27 ± 1.04
	0.5 h UV	25.28 ± 0.97	13.04 ± 0.42
aromatic	2 h UV	13.90 ± 0.29	3.69 ± 0.13
	ethanol soaking	5.17 ± 0.25	2.56 ± 0.17
	heat treatment	29.30 ± 0.63	12.65 ± 2.21
aliphatic	no	7.71 ± 0.13	35.69 ± 0.50
	0.5 h UV	11.20 ± 1.13	34.57 ± 5.36

Additionally, a sample without UV postcuring was tested, where ethanol soaking was used to remove unreacted components. Figure 3d and Table 2 show a sharp decline in the mechanical properties, with an ultimate stress of only 5.17 ± 0.25 MPa and a strain of 2.56 ± 0.17%. This decline is primarily attributed to the low cross-link density resulting from the removal of unreacted monomers, which reduces the plasticizing effect.^13^ Unreacted monomers act as plasticizers, and their removal significantly decreases the fracture strain. An additional hypothesis is that mSLA printing without post-treatment results in lower conversion rates. While high molecular weight polymer chains may form, not all of these chains are covalently connected to the network. These un-cross-linked chains can act as effective cross-links through entanglement, contributing to the overall cross-link density and increasing the ultimate stress.^13^ However, ethanol soaking selectively removes un-cross-linked oligomeric chains, leading to a further reduction in both ultimate tensile strength and strain at break. Beyond this leaching effect, ethanol can also induce microstructural destabilization by swelling the polymer network. Such swelling generates localized stresses, which in turn promote the formation of microcracks and interfacial defects, thereby accelerating mechanical deterioration and compromising the integrity of the printed material. This indicates that unreacted components remain after printing, and UV postcuring is essential for achieving mechanically robust prints.

Despite UV postcuring, the C=C bond signal from methacrylate does not fully disappear, indicating incomplete reaction of unreacted components. To address this limitation, heat treatment was applied to ensure complete polymerization. The sample subjected to 0.5 h of UV postcuring followed by overnight heat treatment at 100 °C demonstrated enhanced mechanical properties, achieving an ultimate tensile strength of 29.30 ± 0.63 MPa and a strain of 12.65 ± 2.21% (Figure 3d). This represents a slight improvement compared to the sample that underwent only 0.5 h of UV postcuring (Table 2, 25.28 ± 0.97 MPa and 13.04 ± 0.42% strain). The improvement in mechanical properties suggests that heat treatment promotes additional cross-linking of the polymer network. The increased network mobility during thermal postcuring allows for further reaction of dangling and uncured chains, which may not be fully cross-linked under UV postcuring at room temperature.^14^ Notably, the heat-treated sample developed a darker yellow color (Figure S13a,c), possibly resulting from molecular rearrangements during extended heating. To explore the underlying causes of the observed structural changes, polarized optical microscopy (POM) and X-ray diffraction (XRD) analyses were performed to investigate the microscopic structure of the post UV cured films, with and without heat treatment. POM images (Figure S13b,d) reveal that all samples retained the printing pattern without evidence of crystallization, indicating an amorphous structure, which was further confirmed by XRD patterns (Figure S14). Both samples displayed a broad diffraction peak at approximately 18°, with increased intensity after heat treatment. This effect can be attributed to the presence of dangling and uncured chains in samples cured at lower UV intensities. The retro-DA reactions enable polymer chains to rearrange at elevated temperatures, which, in turn, increases the mobility of the polymer network during thermal postcuring. This enhanced mobility facilitates additional reactions (polymerization from dangling and uncured chains), contributing to improved cross-linking and enhanced mechanical properties.^14^ Consequently, the molecular chains become more tightly packed, and localized cross-linking may occur, leading to increased material rigidity and strength. These findings underscore the critical role of heat treatment in complementing UV postcuring to achieve complete cross-linking, improved mechanical performance, and enhanced material stability.

To confirm the occurrence of the retro-DA reaction, ATR-FTIR measurements were conducted at various temperatures (Figure 3c). As the temperature increased from 20 to 140 °C, a redshift in the absorbance at approximately 3500 cm^–1^ indicated a minor weakening of intermolecular forces (hydrogen bonding),^7^ whereas the intensification of the furan ring breathing peak at 1020 cm^–1^ confirmed that retro-DA reactions occurred at elevated temperatures.^15^

The performance of the aliphatic DA-based ink (Photoresin 2 prepared from Mixture 2 and BAPO) after postcuring was investigated as well. In contrast to the previously used BSM, this aliphatic bismaleimide (BMI-689) is derived from biomass, significantly increasing the renewable carbon content of biobased ink. The thermal stability of the print from Photoresin 2 results in two major degradation processes, similar to those of the print from Photoresin 1 (Figures 3b and 4a). However, for the print from Photoresin 2, the majority of the material degrades during the first degradation process, where the main polymer chains undergo scission, leaving only a small fraction with high thermal stability (aromatic rings and homopolymerization product from maleimide groups) for the second stage. This observation can be explained by the lower maleimide content, which results in fewer free maleimide groups being released upon retro-DA reaction. The reduced availability of free maleimide promotes homopolymerization instead of efficient reversible cross-linking, leading to a less thermally stable network.^16^

**Figure 4:**
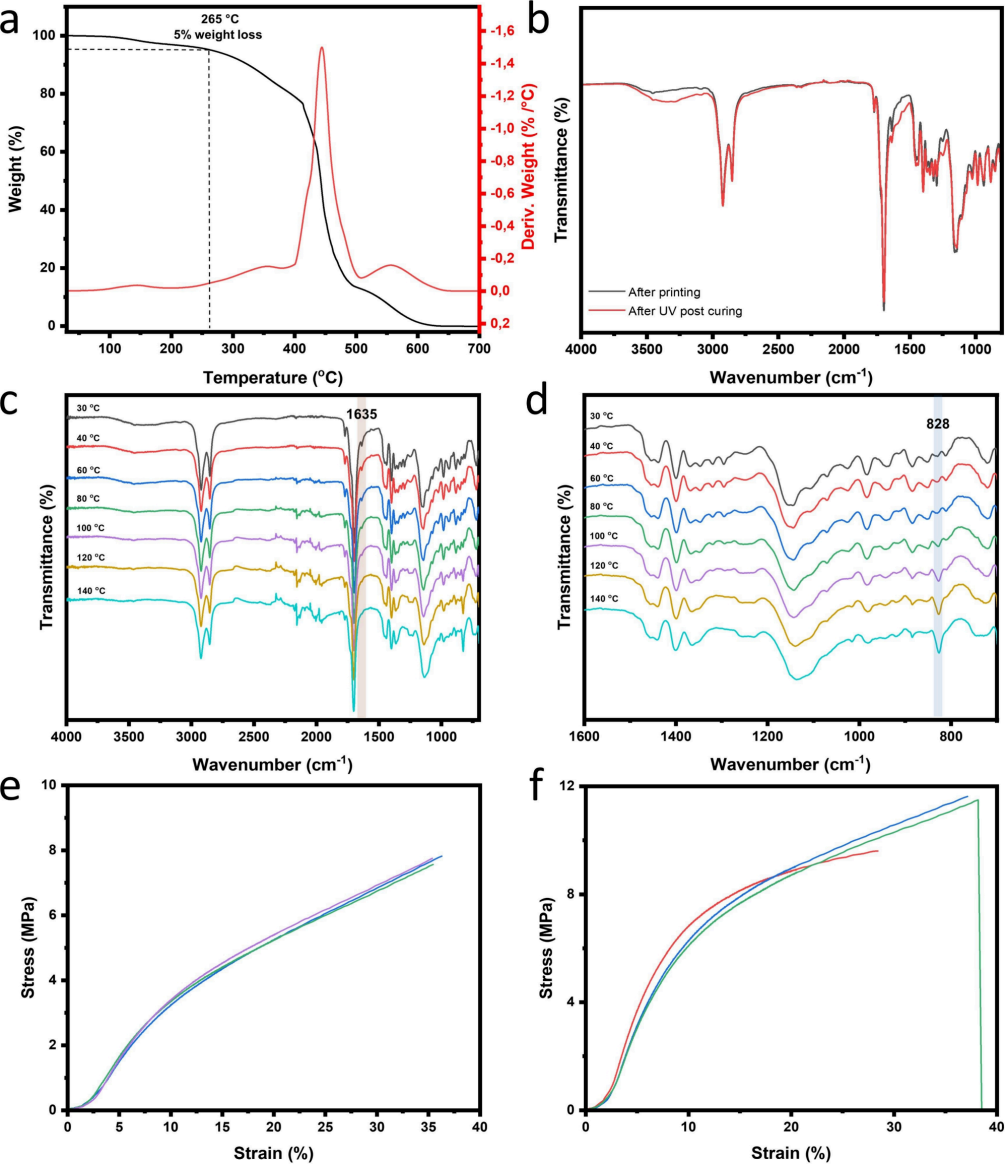
(a) TGA and DTG curves of the sample printed from an aliphatic furan-based film after UV irradiation for 0.5 h; (b) ATR-FTIR spectra of the aliphatic furan-based printed film before and after UV curing; (c) ATR-FTIR spectra of the printed aliphatic thin film in the temperature range of 20 °C to 140 °C; (d) Zoomed-in spectra from 1600 cm^–1^ to 700 cm^–1^ for characteristic absorbance; The stress–strain curves of the printed aliphatic films; (e) Without any post treatment (color lines represent three repetitions); (f) After UV postcuring for 0.5 h (color lines represent three repetitions).

To better understand how residual methacrylate groups influence both the thermal response and the mechanical properties of the printed samples, we monitored the absorbance changes in the IR spectra before and after UV postcuring. As shown in Figure 4b,c, the absorbance at 1635 cm^–1^, which corresponds to the C=C stretching vibrations of unreacted methacrylate groups, gradually diminishes during heating from 30 to 140 °C and eventually vanishes. This indicates that the remaining methacrylate moieties continue to polymerize under external heat, thereby contributing to further network development beyond the initial UV curing stage. Consistent with this spectroscopic evidence, the stress–strain curves presented in Figure 4e,f, together with the data summarized in Table 2, reveal that UV postcuring enhances the ultimate tensile strength from 7.71 ± 0.13 MPa to 11.20 ± 1.13 MPa, whereas the strain remained largely unchanged, mainly owning to the considerable unreacted moieties still served as plasticizers. Furthermore, the retro-DA process was monitored via ATR-FTIR (Figure 4d). The signal at 828 cm^–1^, corresponding to the out-of-plane bending of olefinic protons from maleimide, intensified as the temperature increased, confirming the occurrence of the retro-DA reaction.^17^

To validate the printing accuracy, we employed the aforementioned two photoresins to fabricate a series of complex hollow geometries. As depicted in Figure 5, Photoresin 1 demonstrated high accuracy in printing intricate designs such as a hollow lantern, cube, fullerene, and dog bone spline (Figure 5a–c and e). Similarly, the aliphatic DA-based resin achieved precise printing results, as shown in Figure 5d,f. A notable distinction between the two resins is that the aliphatic DA resin has a lighter color than the aromatic resin does, primarily because BMI-689 has a lighter hue than the aromatic BSM (Figure S15).

**Figure 5:**
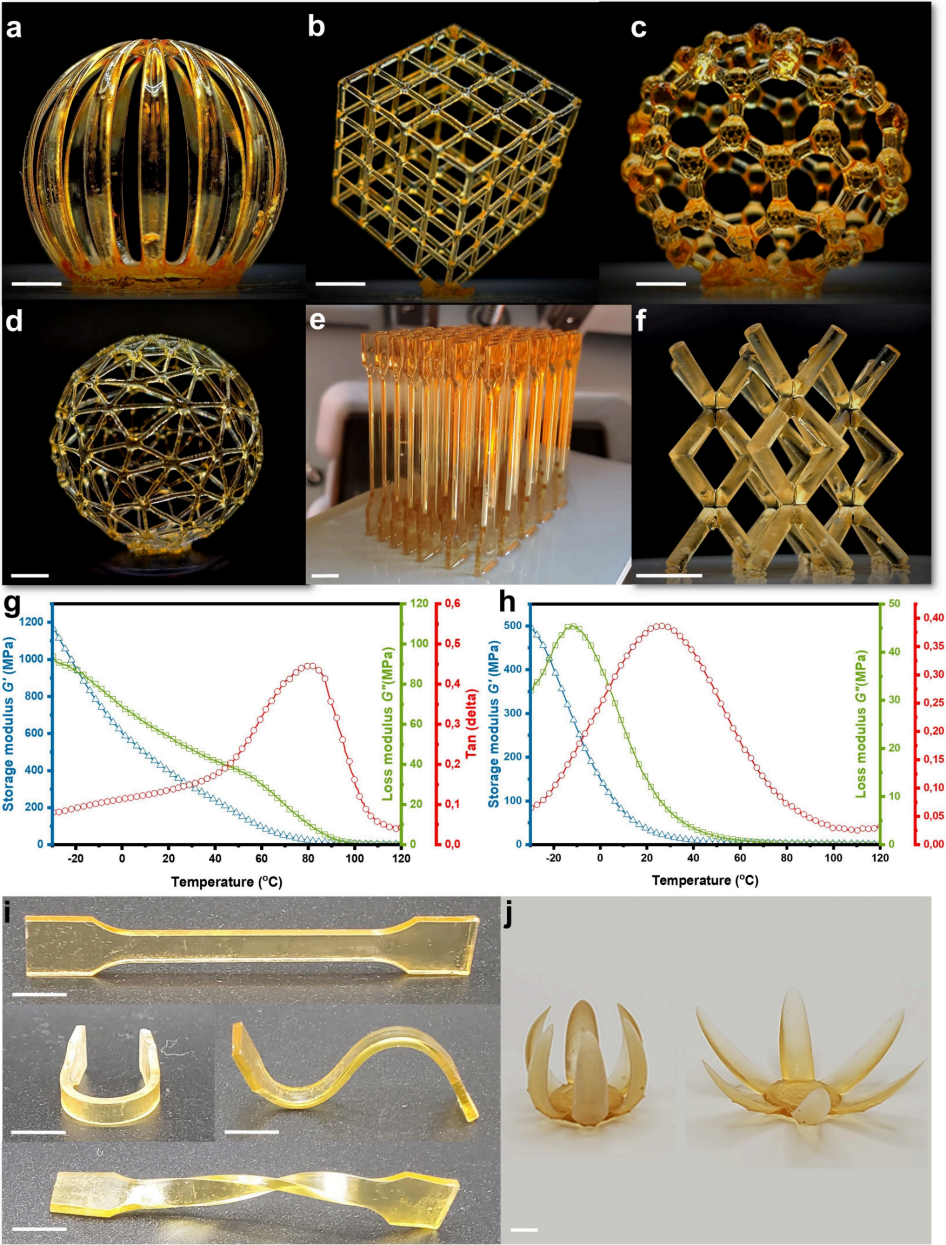
Various hollow structures 3D printed with furan-based resin (Photoresin 1): (a) lanterns; (b) cube; (c) fullerene; (d) ball; (e) tensile stress spline; (f) scaffold; Scale bar, 5 mm. Storage modulus, loss modulus, and loss factor curves for a printed (g) aromatic film and (h) aliphatic film; (i) Visual representation of shape reconfiguration of a printed spline; Scale bar, 5 mm. (j) Flowers printed from Photoresin 1 demonstrating shape memory properties.

Owing to the nature of the Diels–Alder reversible bonds in the DA polymer, these materials are often associated with shape memory properties. Thermally induced shape reprogramming and memory retention are hallmark features of polymeric materials containing dynamic covalent bonds (DCBs).^18^ DCB polymers exist in a glassy state below their *T*_g_, where the motion of molecular segments is restricted, and the material retains its shape. Upon heating above *T*_g_, the molecular segments gain mobility, activating the reversible nature of the dynamic covalent bonds. This enables the material to return to its original shape, driven by internal stress and bond reorganization.^2,19^ In this regard, the *T*_g_ of both samples printed from Photoresins 1 and 2 was measured. As illustrated in Figure 5g,h, the *T*_g_ of the Photoresin 1 printed material is approximately 80 °C, whereas the *T*_g_ of the printed object from Photoresin 2 is approximately 30 °C. Shape memory, which is advantageous in most 3D printing applications, depends on the mobility of the polymer chains when the temperature exceeds the *T*_g_. Owing to the relatively low *T*_g_ of the print from Photoresin 2 (close to room temperature), maintaining a stable shape under ambient conditions is difficult. However, Photoresin 1 exhibits excellent shape retention and can be programmed into various configurations, such as bent, twisted, or spiral shapes, as illustrated in Figure 5i. The shape memory properties were further confirmed through visual demonstrations via printed splines and flowers, as shown in Supplementary Videos 1 and 2. For example, a printed flower was heated above 80 °C via a heat gun, mechanically closed under external stress, and allowed to cool to room temperature, resulting in a temporarily closed shape (Figure 5j). The same protocol was applied to a bent spline. The only difference in procedure was that the flower was reheated with 90 °C hot water, whereas the spline was reheated on a hot plate. Supplementary Video 2 shows the flower returning to its original open shape when immersed in 90 °C hot water.

The sustainability of photoresin preparation process was evaluated via the atom economy (AE), reaction mass efficiency (RME), E-factor, and EcoScale, as summarized in Table 3. This assessment focuses on the entire process, from the DA reaction to the facilitation of the final photo printable ink. Although the conversion rate of the reactants for the DA reaction ranges from 84.1% to 93.4%, the unreacted monomer can still contribute to dilution and plasticization in the subsequent printing process, without generating any waste. Based on this, we consider the AE value to be 100%. The RME value, calculated as the product of the reaction yield and AE, is therefore consistent with the yield across all resins. Furthermore, the preparation process generates no waste, as excess reactants can be used in the subsequent printing process, resulting in an E factor of zero for all formulations. The EcoScale,^9^ a comprehensive metric for evaluating the sustainability of chemical processes, assigns higher scores to greener and more sustainable reactions. All the resins demonstrated desirable EcoScale values (exceeding 75). Deductions in EcoScale scores are attributed to the cost of the FMA monomer, the long reaction time required for the DA reaction, and the toxicity and environmental impacts associated with the BSM monomer. Higher reaction yields correlate with higher EcoScale values. To overcome limitations in EcoScale scores, replacing BSM with aliphatic BMI-689 offers a significant improvement. This substitution enhances safety and environmental compatibility, contributing an additional 10 points to the EcoScale. Consequently, this adjustment maximizes EcoScale values and reinforces the sustainability of photoprintable resins for 3D printing applications. In addition to sustainability, the mechanical properties of the printed material can be improved by introducing intermolecular forces and chemical bonds (e.g., metal coordination bonds and ionic bonds) into the ink formulations.

**Table 3:** Quantitative Sustainability Metrics for the Synthesized Furan Derivatives, Evaluated via the Atom Economy (AE), Reaction Mass Efficiency (RME), E-Factor, and EcoScale

	sustainability metrics				
resin	yield (%)	AE (%)	RME (%)	*E*-factor	EcoScale
R2	84.1	100	84.1	0	76
R5	85.9	100	85.9	0	75
R6	84.7	100	84.7	0	76
R7	90.1	100	90.1	0	79
R8	93.4	100	93.4	0	80

## Conclusions

This study provides a comprehensive investigation into the synthesis, postcuring process, and shape memory behavior of novel DA-based photoresins using a solvent-free and purification-free protocol, with a focus on the synthetic path toward green additive manufacturing. The reaction kinetics of the cycloaddition between FMA and BSM were found to be strongly influenced by the choice of diluent and the furan-to-maleimide molar ratio. Optimal reaction yields were achieved with a 1.1:1 molar ratio using HEMA as the reactive diluent, while PEGMA contributed to enhanced flexibility in the printed structures. Mechanical testing demonstrated that UV postcuring played a crucial role in increasing the ultimate tensile strength, whereas additional heat treatment at 100 °C further improved the material properties through retro-DA reactions, promoting molecular rearrangement and cross-linking. Conversely, prolonged UV exposure and solvent treatments negatively affect mechanical integrity by reducing the cross-link density. Comparisons between Photoresin 1 and 2 revealed that the biomass-derived aliphatic resin improved sustainability without compromising thermal or mechanical performance. Notably, the aromatic resin exhibited superior shape programmability, retention, and thermally induced shape memory properties. These results offer valuable insights into optimizing DA-based resins for sustainable, high-performance 3D printing, particularly in applications that demand mechanical durability and shape memory functionality, such as smart medical devices, actuators, and foldable structures for aerospace. Beyond the scope of DA chemistry, this strategy inspires the exploration of additional in situ photoresin within reactive diluents, opening new avenues for designing environmentally responsible resins compatible with vat photopolymerization.

## Supplementary Material







## Notes

^1^H NMR spectra of the precursors and photoink mixture, digital photo of bismaleimide chemicals, POM image, and XRD curves of cured film (PDF)
Supporting video 1 (MP4)
Supporting video 2 (MP4)

## References

[1] Thorbole A., Bodhak C., Sahu P., Gupta R. K. (2024). Green ink revolution:
Pioneering eco-friendly 3D printing with vegetable oil-derived inks. Polym. Eng. Sci..

[2] Li X., Yu R., He Y., Zhang Y., Yang X., Zhao X., Huang W. (2020). Four-dimensional printing of shape
memory polyurethanes with high
strength and recyclability based on Diels-Alder chemistry. Polymer.

[3] Durand-Silva A., Cortés-Guzmán K. P., Johnson R. M., Perera S. D., Diwakara S. D., Smaldone R. A. (2021). Balancing Self-Healing and Shape
Stability in Dynamic Covalent Photoresins for Stereolithography 3D
Printing. ACS Macro Lett..

[4] Calderon-Ardila S., Morvan D., Péruch O., Bellière-Baca V., Dusselier M., Sels B. F. (2024). Methionine and its
hydroxy analogues:
the paths toward their sustainable chemical synthesis. Green Chem..

[5] Miao J.-T., Yuan L., Liang G., Gu A. (2019). Biobased bismaleimide
resins with high renewable carbon content, heat resistance and flame
retardancy via a multi-functional phosphate from clove oil. Materials Chemistry Frontiers.

[6] Yang B., Ni T., Wu J., Fang Z., Yang K., He B., Pu X., Chen G., Ni C., Chen D., Zhao Q., Li W., Li S., Li H., Zheng N., Xie T. (2025). Circular 3D
printing of high-performance photopolymers through dissociative network
design. Science.

[7] Zhang T., Pacella G., Chen K., Ye T., Portale G., Voet V. S. D., Folkersma R., Loos K. (2025). 3D-Printed Dual Photo-
and Thermally Responsive Materials for Smart Adaptability. Macromolecules.

[8] Thys M., Brancart J., Van Assche G., Van den Brande N., Vendamme R. (2023). LignoSwitch: A Robust Yet Reversible
Bioaromatic Superglue
for Enhanced Materials Circularity and Ecodesign. ACS Sustainable Chem. Eng..

[9] Van
Aken K., Strekowski L., Patiny L. (2006). EcoScale, a semi-quantitative tool
to select an organic preparation based on economical and ecological
parameters. Beilstein journal of organic chemistry.

[10] Post C., Maniar D., Jongstra J. A., Parisi D., Voet V. S. D., Folkersma R., Loos K. (2024). Enzymatic bulk synthesis, characterization,
rheology, and biodegradability of biobased 2,5-bis­(hydroxymethyl)­furan
polyesters. Green Chem..

[11] Jiang Y., Hadjichristidis N. (2021). Diels–Alder
Polymer Networks with Temperature-Reversible
Cross-Linking-Induced Emission. Angew. Chem.,
Int. Ed..

[12] Štaffová M., Ondreáš F., Svatík J., Zbončák M., Jančář J., Lepcio P. (2022). 3D printing and post-curing optimization of photopolymerized
structures: Basic concepts and effective tools for improved thermomechanical
properties. Polym. Test..

[13] Uzcategui A. C., Muralidharan A., Ferguson V. L., Bryant S. J., McLeod R. R. (2018). Understanding
and Improving Mechanical Properties in 3D printed Parts Using a Dual-Cure
Acrylate-Based Resin for Stereolithography. Adv. Eng. Mater..

[14] Anastasio R., Peerbooms W., Cardinaels R., van Breemen L. C. A. (2019). Characterization
of Ultraviolet-Cured Methacrylate Networks: From Photopolymerization
to Ultimate Mechanical Properties. Macromolecules.

[15] Wang S., Wang N., Kai D., Li B., Wu J., Yeo J. C. C., Xu X., Zhu J., Loh X. J., Hadjichristidis N., Li Z. (2023). In-situ forming dynamic covalently
crosslinked nanofibers with one-pot closed-loop recyclability. Nat. Commun..

[16] Alrefai M., Maric M. (2025). Self-Healing Biobased Thermoreversible
Polymer Networks by Photo-Diels-Alder
Chemistry. J. Polym. Sci..

[17] Peterson A., Roy M., Fagerlund J., Lo Re G., Müller C. (2021). Synergistic
reinforcement of a reversible Diels–Alder type network with
nanocellulose. Materials Advances.

[18] Zheng N., Fang Z., Zou W., Zhao Q., Xie T. (2016). Thermoset
Shape-Memory Polyurethane with Intrinsic Plasticity Enabled by Transcarbamoylation. Angew. Chem., Int. Ed..

[19] Choi S., Park B., Jo S., Seo J. H., Lee W., Kim D.-G., Lee K. B., Kim Y. S., Park S. (2022). Weldable and
Reprocessable Shape Memory Epoxy Vitrimer Enabled by Controlled Formulation
for Extrusion-Based 4D Printing Applications. Adv. Eng. Mater..

