# Supplementary material for: In Situ Photoresin Synthesis via Reactive Diluents for Vat Photopolymerization
Source: Biomacromolecules. 2025 Oct 9;26(11):8017–26. doi: 10.1021/acs.biomac.5c01471 (PMC12606631; doi:10.1021/acs.biomac.5c01471)
Supplement: Supplementary file 1 [file bm5c01471_si_001.pdf]

In situ Photoresin Synthesis via Reactive Diluents for Vat Photo-polymerization

Tao Zhang[1,2], Vincent S. D. Voet[2], Rudy Folkersma[2] and Katja Loos[1]

[1]Macromolecular Chemistry and New Polymeric Materials, Zernike Institute for Advanced Materials, University of Groningen, Nijenborgh 3, Groningen 9747 AG, The Netherlands

[2]Circular Plastics, Academy Tech & Design, NHL Stenden University of Applied Sciences, Van Schaikweg 94, 7811 KL, Emmen, The Netherlands

Table of Contents

HEMA ^{1}H NMR spectrum 2
BSM ^{1}H NMR spectrum 3
FMA ^{1}H NMR spectrum 4
PEGMA ^{1}H NMR spectrum 5
BMI-689 ^{1}H NMR spectrum 6
R1 resin ^{1}H NMR spectrum 7
R2 resin ^{1}H NMR spectrum 8
R6 resin ^{1}H NMR spectrum 9
R7 resin ^{1}H NMR spectrum 10
R8 resin ^{1}H NMR spectrum 11
R5 resin ^{1}H NMR spectrum 12
DA based resin from FMA and BMI-689 ^{1}H NMR spectrum 13
Digital photo and POM images 14
XRD curve 15
Digital photo of BSM and BMI-689 16

HEMA ^{1}H NMR spectrum

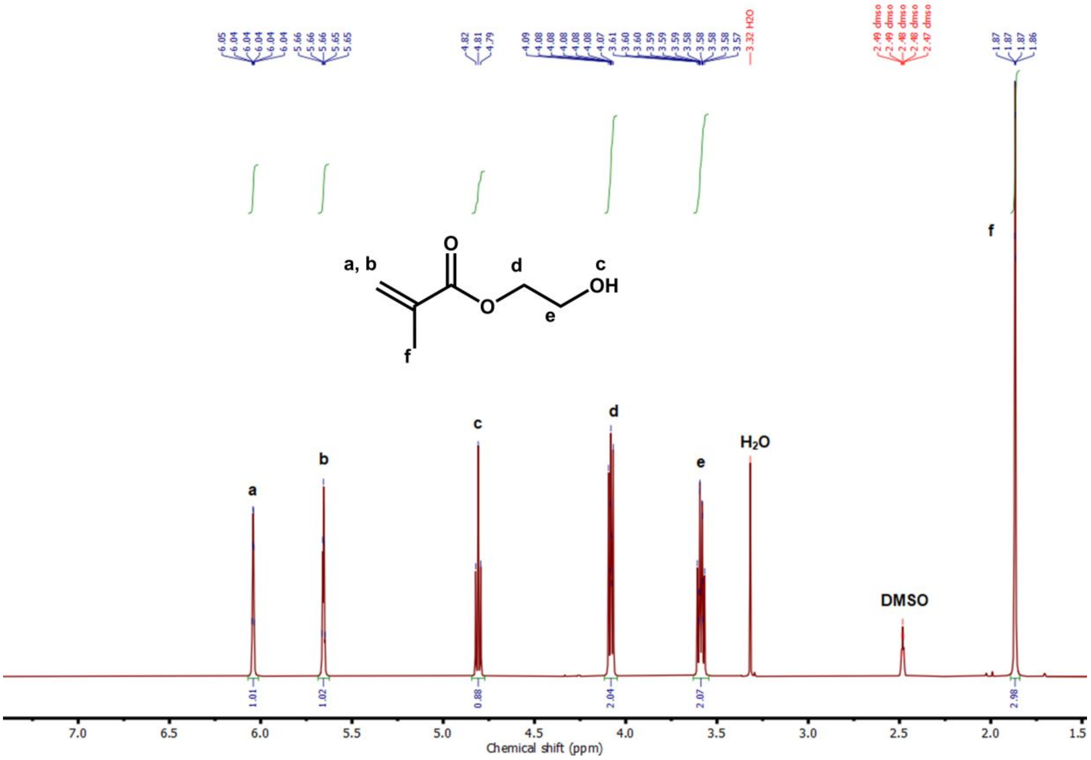

Figure S1. ^{1}HNMR spectrum of 2-Hydroxyethyl Methacrylate (HEMA).

BSM ^{1}H NMR spectrum

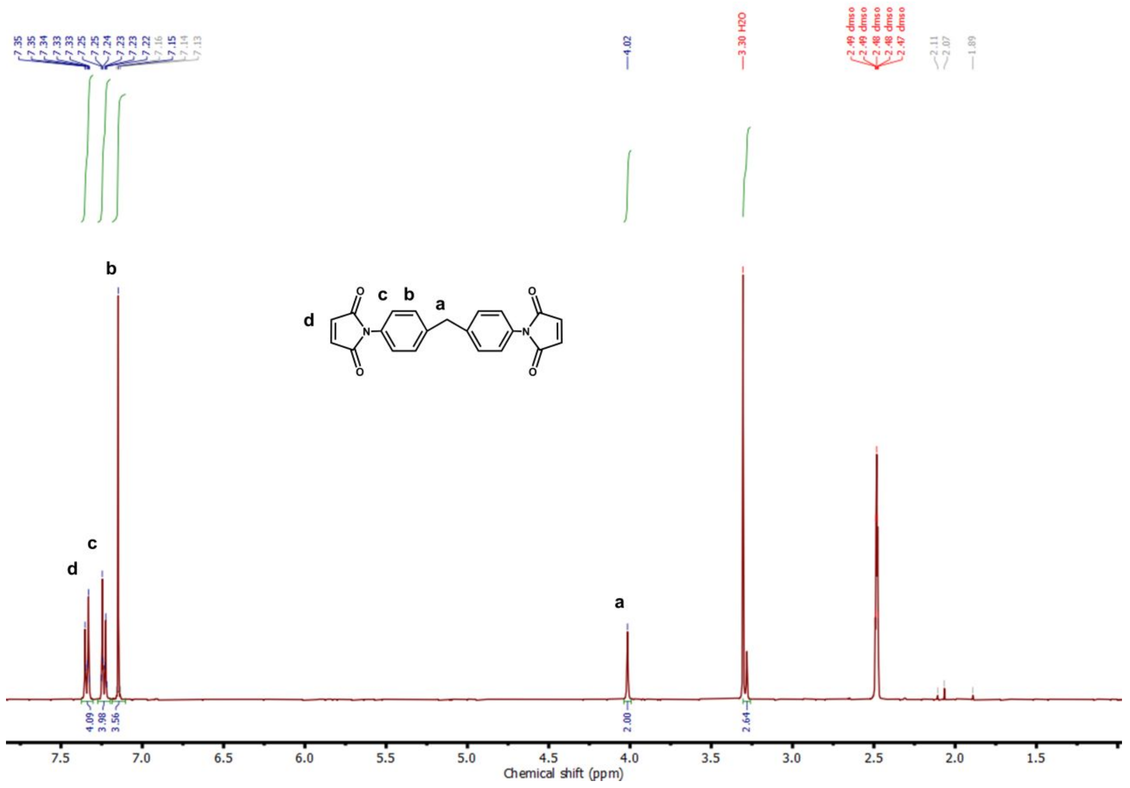

Figure S2. ^{1}HNMR spectrum of 4,4'-Bismaleimidodiphenylmethane (BSM).

FMA ^{1}H NMR spectrum

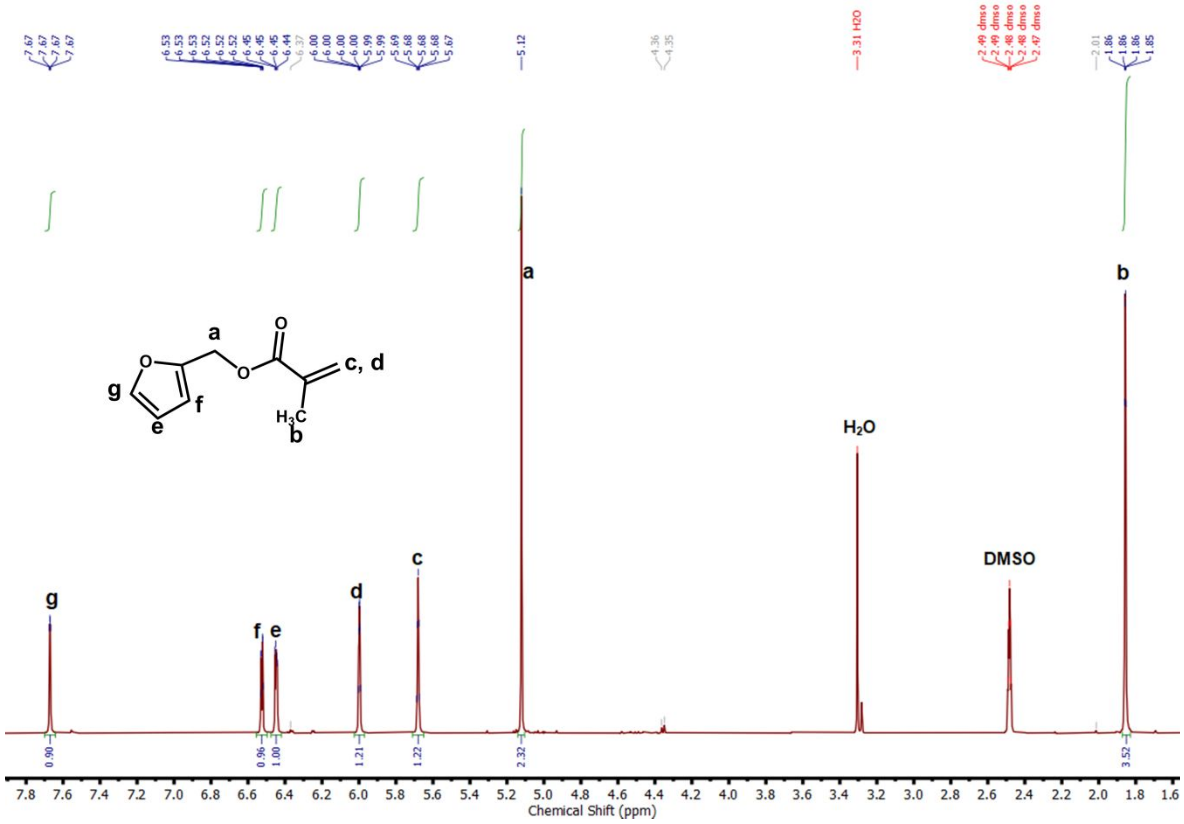

Figure S3. ^{1}HNMR spectrum of furfuryl methacrylate (FMA).

PEGMA ^{1}H NMR spectrum

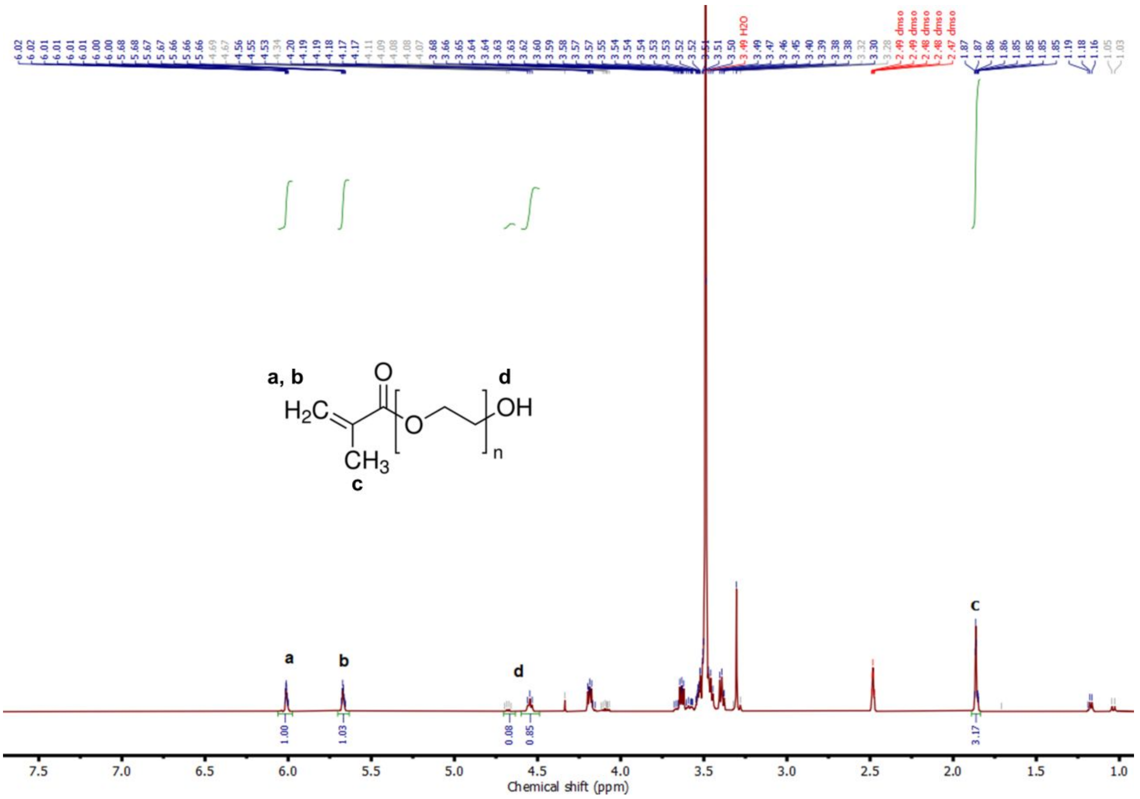

Figure S4. ^{1}HNMR spectrum of Poly(ethylene glycol) methacrylate (PEGMA, Mn 360)

BMI-689 ^{1}H NMR spectrum

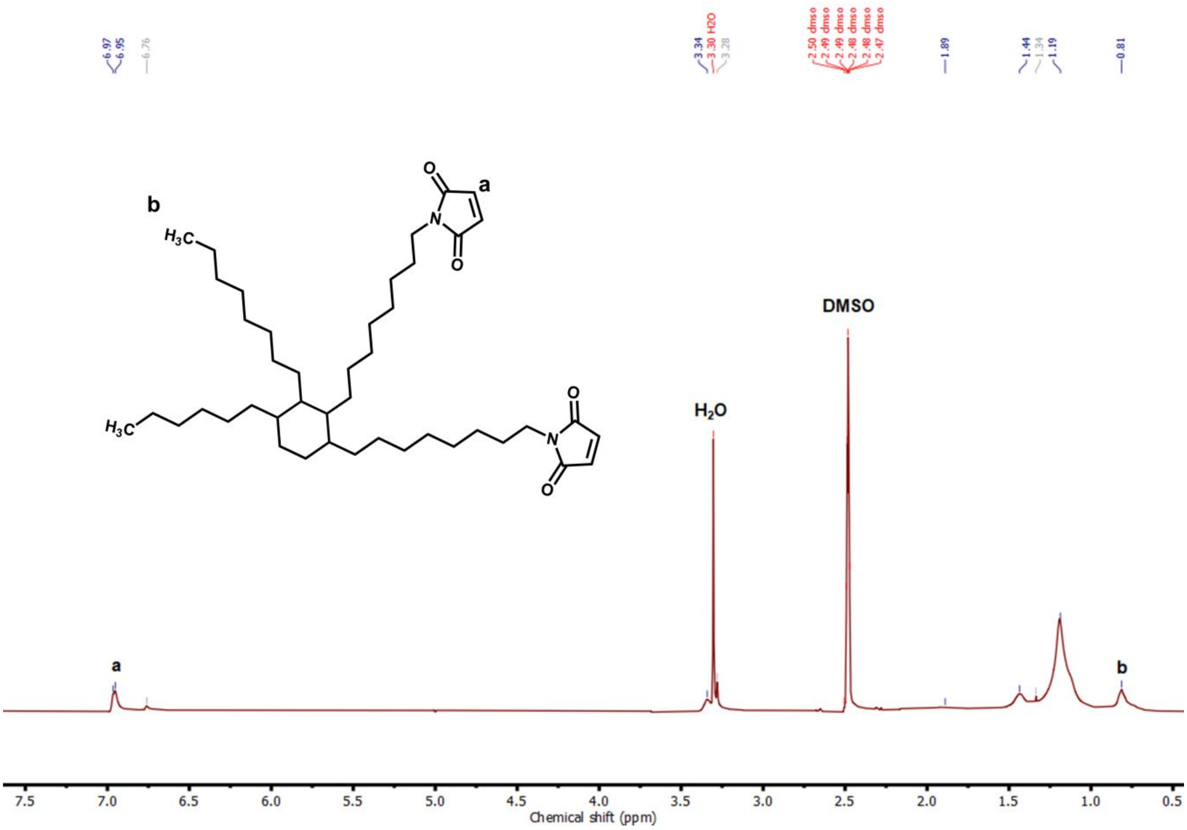

Figure S5. ^{1}HNMR spectrum of BMI-689.

R1 resin ^{1}H NMR spectrum

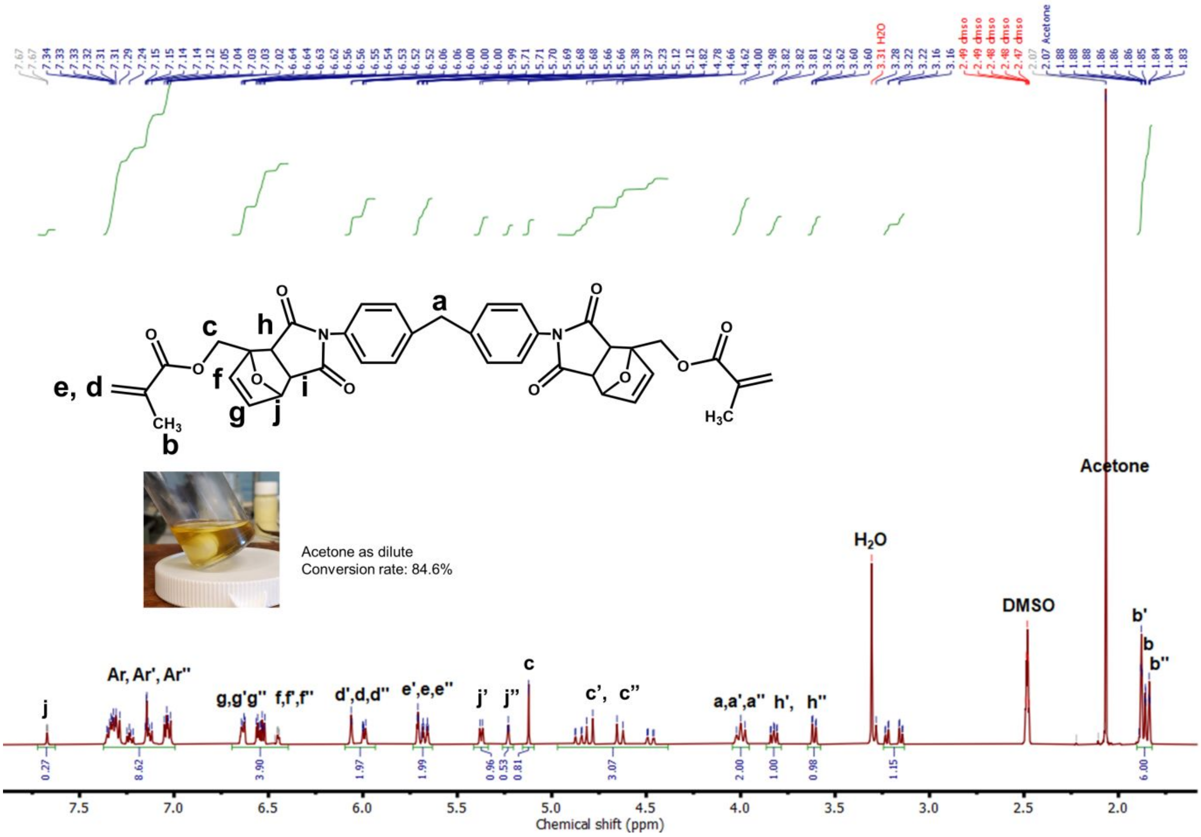

Figure S6. ^{1}H NMR spectrum of the product from an aromatic Diels-Alder reaction, with Acetone as the diluent (DMSO-*d6*).

R2 resin ^{1}H NMR spectrum

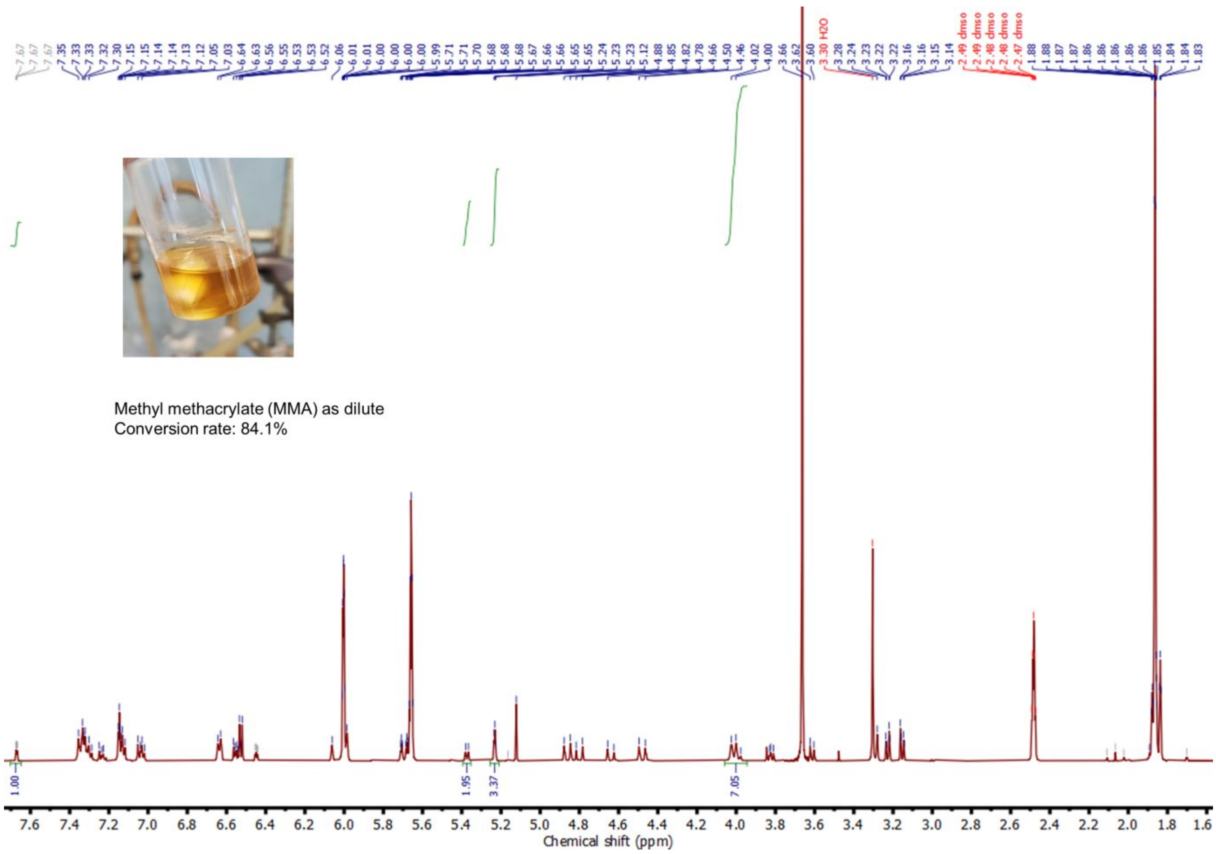

Figure S7. ^{1}H NMR spectrum of the product from an aromatic Diels-Alder reaction, with MMA as the diluent.

R6 resin ¹H NMR spectrum

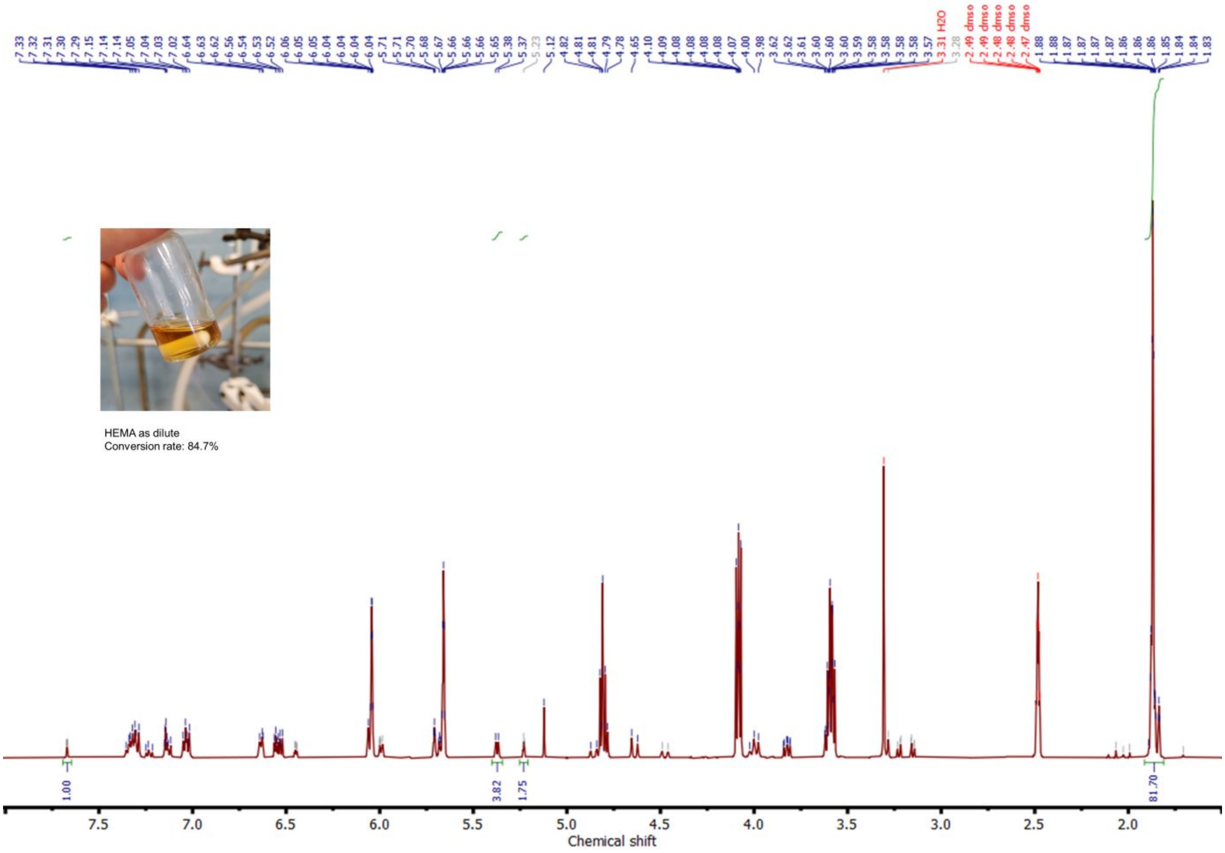

Figure S8. ¹H NMR spectrum of the product from an aromatic Diels-Alder reaction, with HEMA as the diluent.

R7 resin ^{1}H NMR spectrum

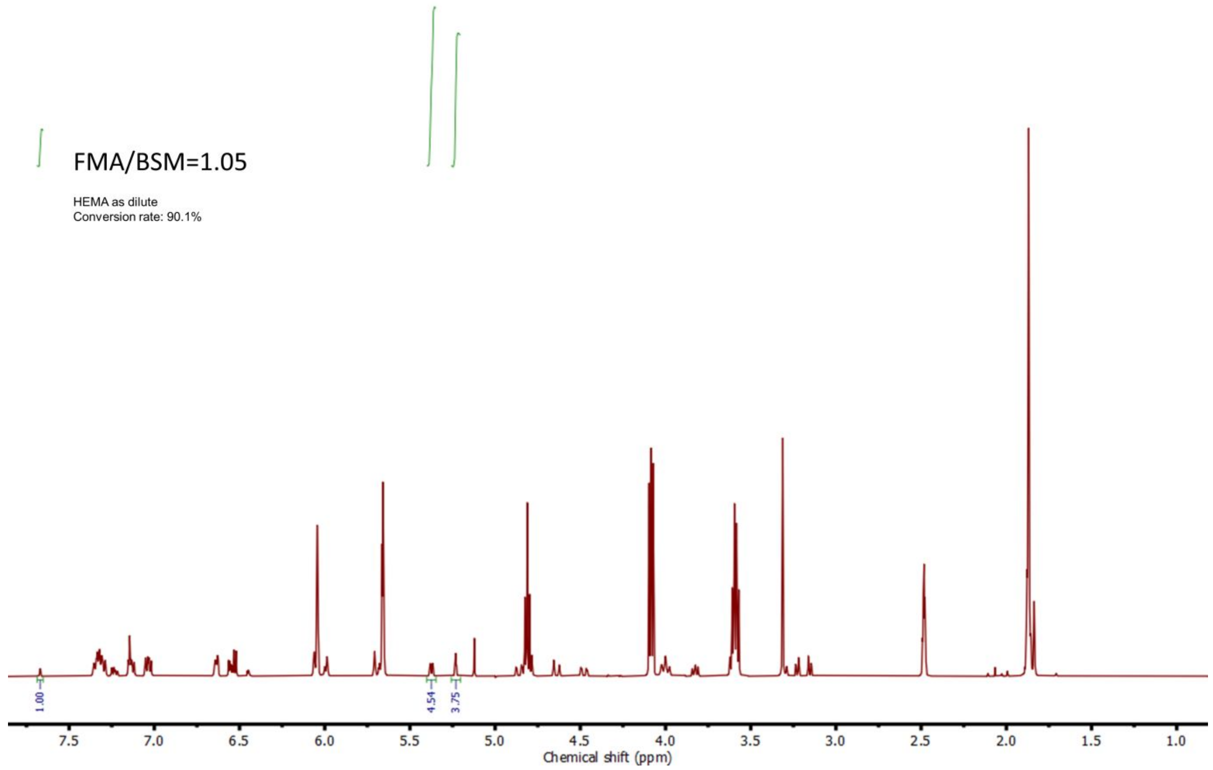

Figure S9. ^{1}H NMR spectrum of the product from an aromatic Diels-Alder reaction, with HEMA as the diluent.

R8 resin ^1H NMR spectrum

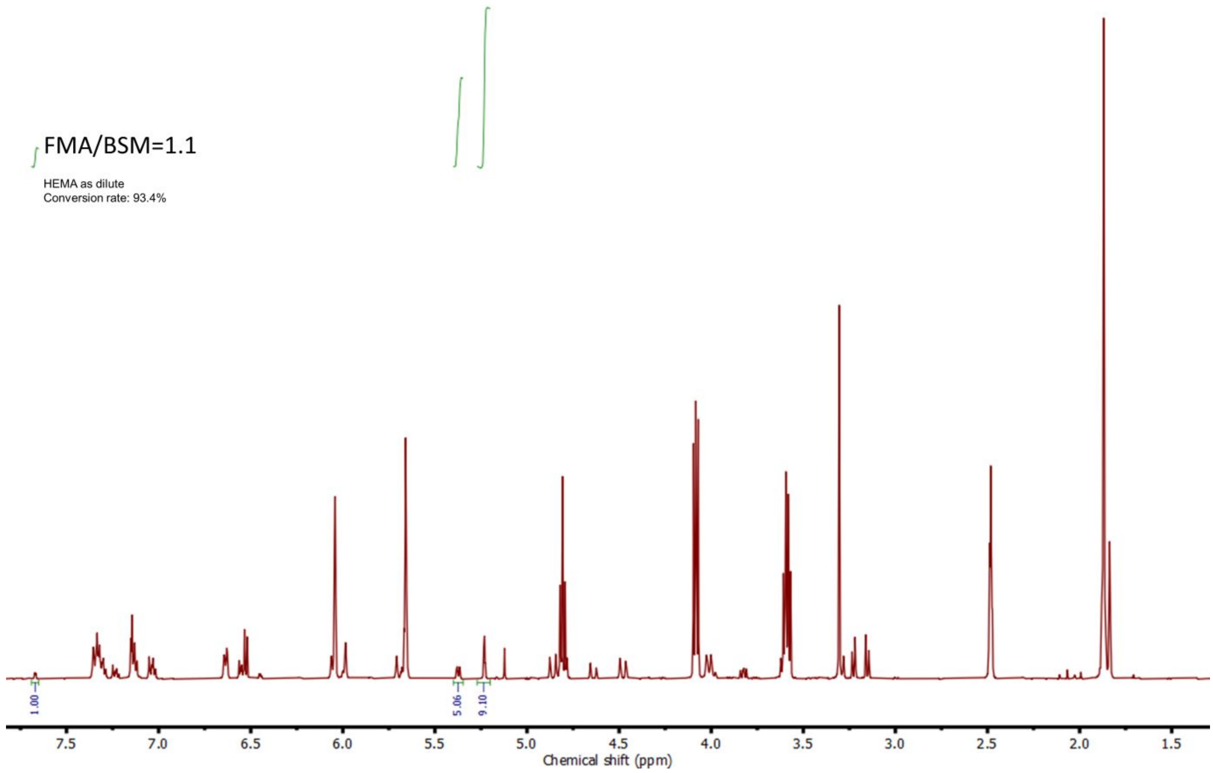

Figure S10. ^1H NMR spectrum of the product from an aromatic Diels-Alder reaction, with HEMA as the diluent.

R5 resin ^{1}H NMR spectrum

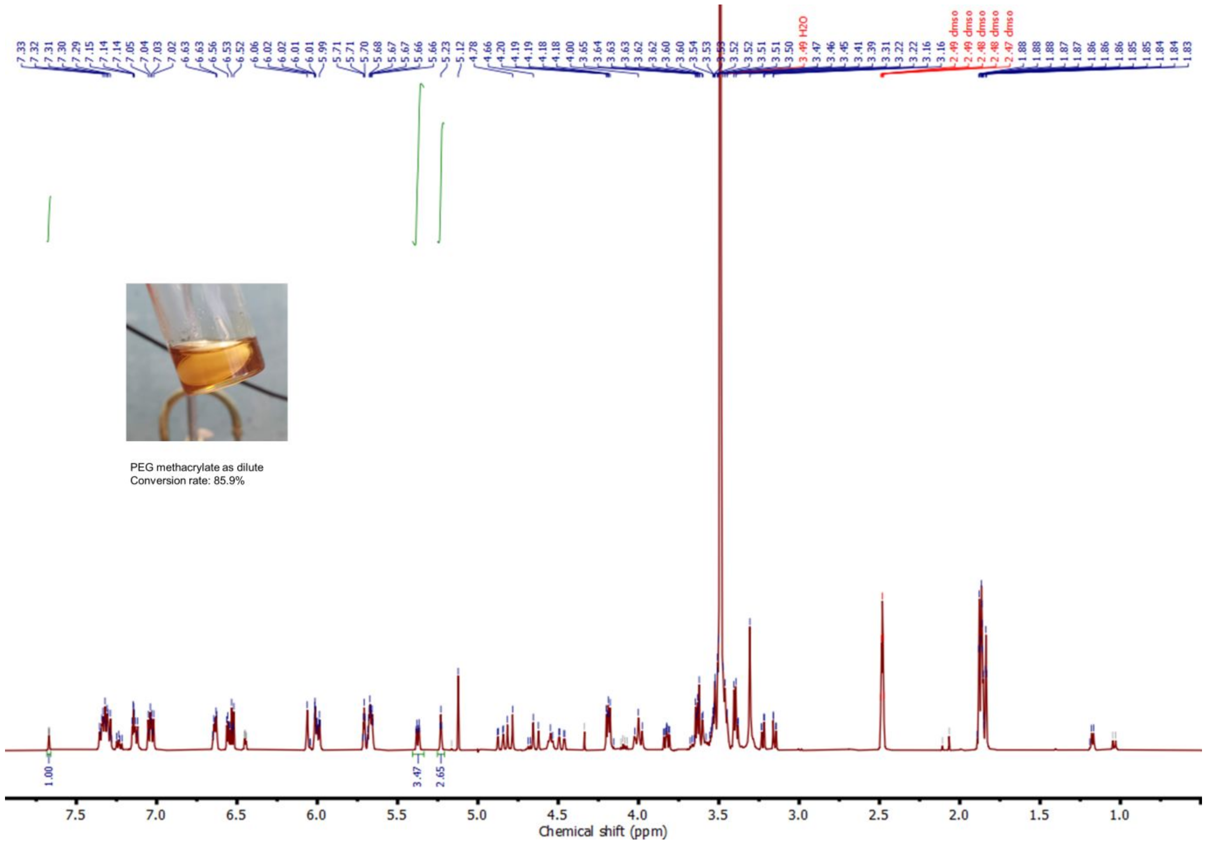

Figure S11. ^{1}H NMR spectrum of the product from an aromatic Diels-Alder reaction (FMA/BSM=1), with PEG methacrylate as the diluent.

DA based resin from FMA and BMI-689 ^{1}H NMR spectrum

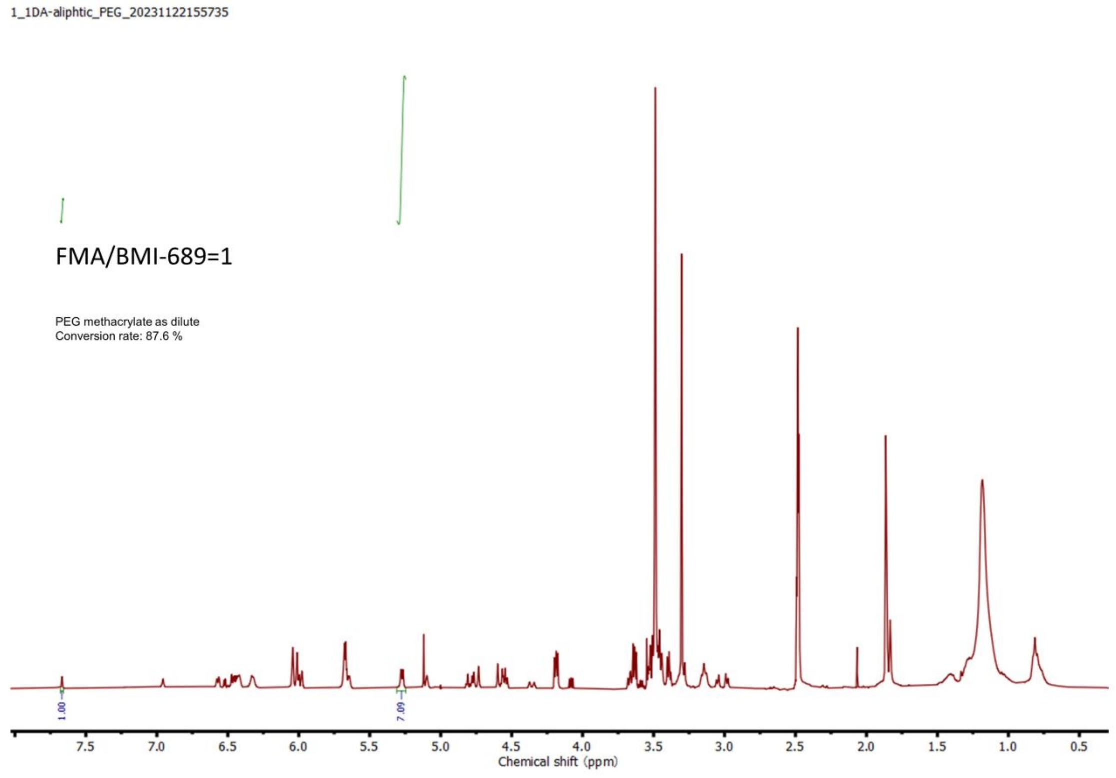

Figure S12. ^{1}H NMR spectrum of the product from an aliphatic Diels-Alder reaction, with PEGMA as diluent.

Digital photo and POM images

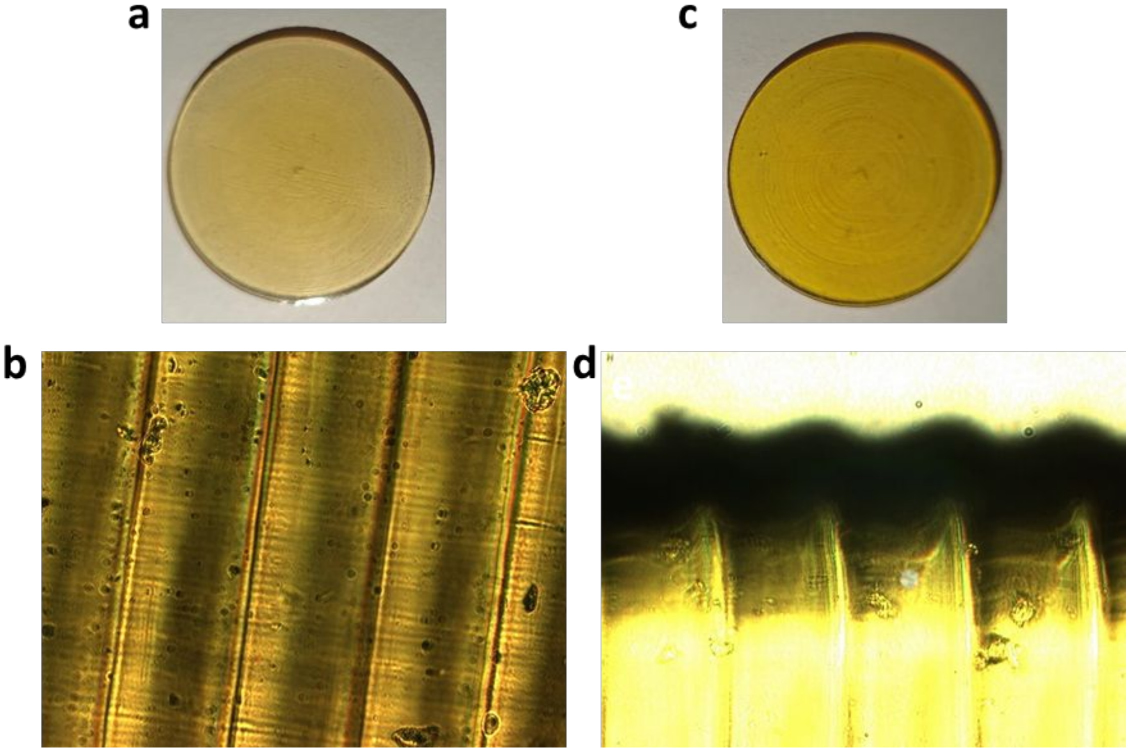

Figure S13. (a) digital photo of DA-aromatic film before 100 ℃ treatment. (b) POM image of DA-aromatic film before 100 ℃ treatment. (c) digital photo of DA-aromatic film after 100 ℃ treatment. (d) POM image of DA-aromatic film after 100 ℃ treatment.

XRD curve

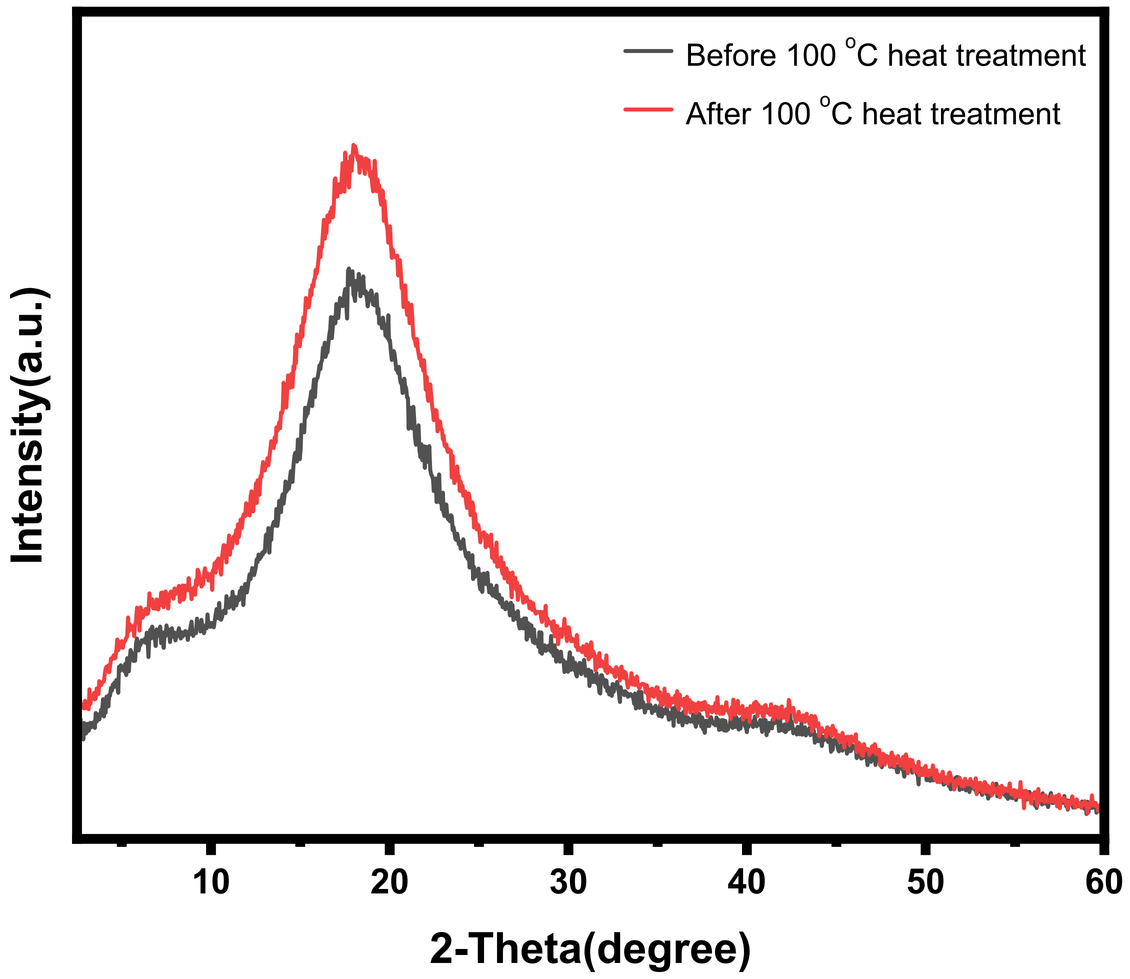

Figure S14. XRD curves of the sample before and after heat treatment at 100 °C.

Digital photo of BSM and BMI-689

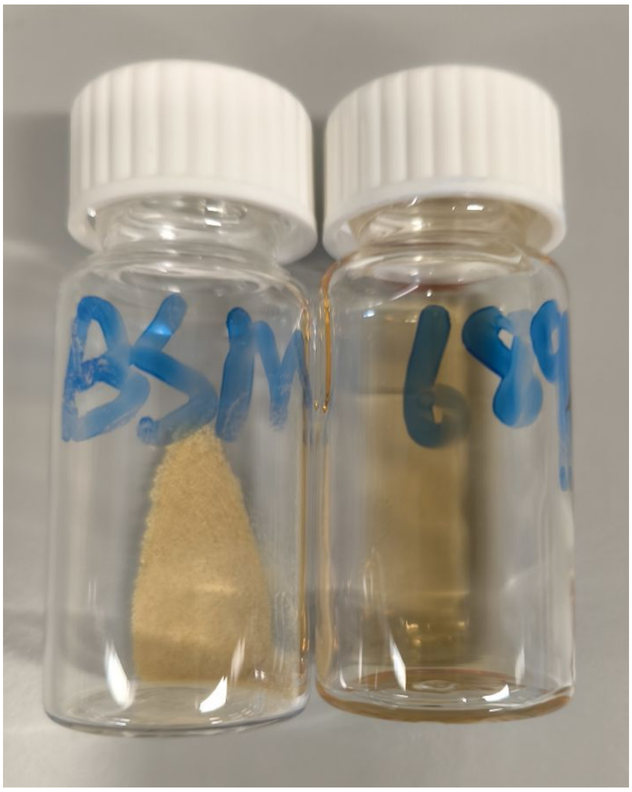

Figure S15. Digital photo of monomer BSM (left) and BMI-689 (right).
